# Reducing antipsychotic drugs in stable patients with chronic schizophrenia or schizoaffective disorder: a randomized controlled pilot trial

**DOI:** 10.1007/s00406-020-01109-y

**Published:** 2020-02-15

**Authors:** Maximilian Huhn, Claudia Leucht, Philipp Rothe, Markus Dold, Stephan Heres, Susanne Bornschein, Thomas Schneider-Axmann, Alkomiet Hasan, Stefan Leucht

**Affiliations:** 1 Department of Psychiatry and Psychotherapy, Klinikum Rechts Der Isar, School of Medicine, Technical University of Munich, Ismaningerstr. 22, 81675 Munich, Germany; 2 Department of Psychiatry, Psychosomatic Medicine and Psychotherapy, Social Foundation Bamberg, Teaching Hospital of the University of Erlangen, Erlangen, Germany; 3 Department of Forensic Psychiatry and Psychotherapy, Günzburg District Hospital, University of Ulm, Ludwig-Heilmeyer-Straße 2, Guenzburg, Germany; 4 Department of Psychiatry and Psychotherapy, Medical University of Vienna, Währinger Gürtel 18-20, Vienna, Austria; 5 Klinik Nord, Isar-Amper-Klinikum München Ost, Munich, Kölner Platz, Haus 7, 80804 Munich, Germany; 6 Private Practice for Psychiatry and Psychotherapy, Nymphenburger Str. 139, 80636 Munich, Germany; 7 Inn-Salzach-Klinikum Wasserburg Am Inn, Gabersee 7, 83512 Wasserburg Am Inn, Germany; 8 Department of Psychiatry and Psychotherapy, University Medical Hospital, Nußbaumstr. 7, 80336 Munich, Germany; 9 Department of Psychiatry, Psychotherapy and Psychosomatics of the University Augsburg, Bezirkskrankenhaus Augsburg, Augsburg, Germany

**Keywords:** Randomized clinical trial, Antipsychotic maintenance treatment, Dose reduction, Chronic schizophrenia, Relapse

## Abstract

As the course of schizophrenic disorders is often chronic, treatment guidelines recommend continuous maintenance treatment to prevent relapses, but antipsychotic drugs can cause many side effects. It, therefore, seems reasonable to try to reduce doses in stable phases of the illness or even try to stop medication. We conducted a 26 weeks, randomized, rater blind, feasibility study to examine individualized antipsychotic dose reduction versus continuous maintenance treatment (Register Number: NCT02307396). We included chronic, adult patients with schizophrenia or schizoaffective disorder, who were treated with any antipsychotic drug except clozapine, who had not been hospitalized in the last 3 years and who were in symptomatic remission at baseline. The primary outcome was relapse of positive symptoms. Symptom severity, social functioning and side effects were also examined as secondary outcomes. 20 patients were randomized. Relapse rates in the two groups were not significantly different. No patient had to be hospitalized. One patient in the control group dropped out. The mean reduction of antipsychotic dose in the individualized dose-reduction group was 42%, however only one patient discontinued drug completely. There were no significant differences in efficacy or safety outcomes. This randomized trial provides evidence, that reduction of antipsychotic medication in chronic stable schizophrenic patients may be feasible. The results need to be confirmed in a larger trial with a longer follow-up period.

**Electronic supplementary material:**

The online version of this article (10.1007/s00406-020-01109-y) contains supplementary material, which is available to authorized users.

## Introduction

Schizophrenia is a debilitating disorder with a frequently chronic course. Relapses are a common problem in schizophrenia which are not only a heavy burden for patients and their relatives [1], but which also cause high socio-economical costs [2]. Around 80% of patients with a first episode experience another acute episode within 5 years [3]. Therefore, prevention of relapse is a central goal of guideline-based treatment. Antipsychotic drugs reduce relapse rates effectively from 64 to 27% after a year of treatment, as shown in a large meta-analysis [4]. Relapse rates were similar after 3–6 years [4]. Furthermore, a large cohort study has shown that long-term antipsychotic treatment prevents rehospitalization and reduces mortality in first episode patients [5]. Therefore guidelines recommend drug treatment as first option for acute and maintenance treatment of schizophrenia [6, 7]. Nevertheless, reducing antipsychotic doses in chronic schizophrenic patients is an important goal, because antipsychotics produce many side-effects. For example, metabolic side-effects can accumulate over time leading to an increased cardiovascular risk for patients with schizophrenia [8]. Furthermore, studies suggest that treatment with antipsychotics may reduce brain volume in a dose-related fashion [9]. It is therefore important to find out by how much patients can reduce antipsychotic doses or in how many patients antipsychotics can be completely withdrawn. There are three main withdrawal options. First, abrupt stopping of the antipsychotic as often done in typical relapse prevention studies, an approach, which has consistently been shown to increase relapse risk in placebo-treated patients [4]. Second, restarting antipsychotic treatment after complete withdrawal, if prodromal syndromes occur. This so-called intermittent treatment is also associated with higher relapse rates compared to continuous treatment [10]. A third option, often used in clinical routine, is the gradual dose reduction tailored to the individual patient symptoms and needs. The rationale is that there is probably a lot of interindividual variability of the doses needed for relapse prevention. Cohort studies have shown that up to one third of the patients with schizophrenia can stop medication and that these patients had more periods of recovery [11]. In a cohort study with first episode patients 30% had remission of psychotic symptoms with no current use of antipsychotic medication at 10-year follow up [12]. 50% antipsychotic dose reduction did not increase positive symptoms and even improved cognitive function and negative symptoms in two randomized controlled trials [13, 14]. In contrast, a 14-year follow-up study in rural China found (partial) remission rates of 57% in the group taking antipsychotics compared to 30% in a group that did not [15]. Overall many patients want to reduce medication, in particular, if they suffer from severe side effects (e.g. weight gain, sedation) which reduce their quality of life. Therefore, they often reduce doses without professional support. This can lead to severe relapses and sometimes, violent acts or even death due to suicide. Thus, dose reduction seems reasonable and recommendations from the user-movement initiated a general debate on reduction of medication [16, 17], but it probably needs to be carefully adapted to the individual patient´s psychopathological state. Such an approach was first systematically evaluated by Wunderink and colleagues. They tried gradual symptom guided tapering of dosage in stable first episode patients. Relapse rates were 20% higher in discontinued patients, but mostly of mild severity [18]. To examine, if gradual symptom guided dose reduction is possible in remitted chronic schizophrenic patients, we conducted a pilot feasibility study.

## Methods

### Study design and participants

We conducted a single-blind, randomised, controlled pragmatic feasibility trial between November 5, 2014 and 30 July 2016, in the outpatient department of the psychiatry department of the Technical University, Munich, Germany. Eligible participants were between 18 and 65 years old meeting ICD-10 criteria for schizophrenia or schizoaffective disorder. The participants had to be stable for at least 3 years, defined by no psychiatric hospitalization, and they had to be continuously treated with antipsychotic medication with no changes in the last four weeks. There was no restriction in terms of the initially used antipsychotics and their doses, except for the exclusion of clozapine. This antipsychotic is reserved for treatment-resistant patients and assumed to be associated with a high risk for rebound psychoses [19]. Moreover, patients had to be in symptomatic remission of positive symptoms as defined by the following criteria:

1. PANSS-items (positive items of the Andreasen criteria [20]) < 4: delusions (P1), conceptual disorganisation (P2), hallucinations (P3), mannerisms and posturing (G5) and unusual thought content (G9).
2. CGI < 4 [21].

Further exclusion criteria were substance dependence other than tobacco dependency, suicidality, and initiation or dose change of antidepressants or mood stabilizers during the last six weeks before enrolment.

Our trial protocol was approved by the ethics committee of the Technical University of Munich and the trial is registered at clincialtrial.gov with the registration number: NCT02307396. All patients gave written informed consent prior to study inclusion.

### Randomisation and masking

Participants were recruited from outpatient clinics of psychiatric hospitals, ambulant psychiatrists or newsletters to patient self-help groups in the area of Munich, Germany. Eligible participants were then randomized either to the intervention (reducing of medication) or control group (no dose reduction) via a fax from the trial coordinator to A.H. who had the randomization list. The randomization list was generated by an independent psychologist using the RAND function of Microsoft Excel 2010. Only once the patient was recorded, allocation was made known to the treating psychiatrist and the participant. The ratings were done by clinicians who were blind to the allocation (C.L., P.R. and S.B.).

### Procedures

In the intervention group, antipsychotic dose was gradually reduced and stopped if possible, based on the participant’s psychopathological status. As a rule the initial antipsychotic dose should be reduced by 1/6 every other week for the first three months, but this was adapted for each patient individually according to her/his needs and psychopathological status. So antipsychotic doses were reduced as far as possible for the first three months and then patients were followed-up with stable medication for three months. Medication in the control group was maintained. Participants were provided with two tablets of lorazepam 1 mg as rescue medication, which were renewed, if used. Trial duration was 26 weeks with study visits every two weeks. The antipsychotic doses were adjusted by M.H., who treated the patients during the reduction. To ensure adherence to the study protocol, M.H. did a pill count at all study visits and drug levels were measured at baseline, after 12 weeks and at the endpoint. Participants were seen every two weeks and had an emergency phone number which they could call 24 h each day. All participating clinicians (M.H., C.L., P.R. and S.B.) had at least an experience of 6 years in clinical psychiatry.

### Outcomes

#### Primary outcome

The primary outcome was relapse defined as a CGI > 3 AND at least two of the following positive PANSS items > 3: delusions, conceptual disorganisation, hallucinations, mannerisms and posturing and unusual thought content assessed at every visit.

#### Secondary outcomes

The following rating scales were applied:

- “Positive and Negative Syndrome Scale” (PANSS) [22]
- “Subjective well-being under neuroleptics scale (SWN)” [23]
- “Personal and Social Performance Scale (PSP)” [24]
- “Medication Adherence Rating Scale (MARS)” [25]
- “Abnormal Involuntary Movement Scale (AIMS)” [26]

All raters were trained in the PANSS by S.H. We assessed dropouts due to any reason and due to specific reasons. Side effects were measured with the UKU-scale [27]. Olanzapine equivalence doses were calculated using the “International consensus study of antipsychotic dosing” [28].

### Statistical analysis

The intention-to-treat-sample consisted of all participants randomized into the trial. The per-protocol-sample were all participants completing the trial according to protocol.

The primary dichotomous outcome was the number of patients relapsed in the intervention group compared with that in the control group. Fisher’s Exact Test was applied to compare the number of relapsed patients.

As secondary endpoint time until relapse or study endpoint was graphically presented with Kaplan–Meier curves. Group differences in time to relapse were examined with the log-rank test. In addition, the median time to relapse for both study arms was determined. Cox's proportional hazard model (Cox Regression Analysis) was used to determine the effect of treatment parameters age, gender and total PANSS score at baseline (independent variable) on the outcome time to relapse (dependent variable).

Kolmogorov–Smirnov tests on normality were performed. For continuous outcomes, ANOVA was performed to compare data at baseline between the groups, if for a variable there were no significant deviations from normality assumption. Mann–Whitney *U* tests were used, if data were tested not to be normally distributed. In case of dichotomous variables (e.g. gender), group comparisons were performed with Fisher's Exact Test or with Freeman–Halton test for 2 × 3 tables.

The changes in the rating scales [overall PANSS score, CGI-S, quality of life (SW-N), personal and social functional level (PSP), adherence behavior (MARS), scales for the evaluation of movement disorders (AIMS)] from time of randomization until the end of study were calculated by analysis of covariance (ANCOVA) with treatment (discontinuation or continuation of antipsychotic treatment) as a fixed factor and the respective scale value at baseline as a covariate. If the assumptions for parametric tests were violated, Mann–Whitney *U* test or Freeman-Halton test were used as appropriate.

The occurrence of specific side effects (UKU scale) is shown descriptively and compared between the groups with Fisher’s Exact Test. Breslow–Day tests were used to analyze, if the side effects changed significantly different between the groups from baseline until the end of the study. If there were missing values at study endpoint, last observation carried forward (LOCF) procedure was applied.

The primary statistical analysis followed the intention-to-treat (ITT) principle defined as all patients being randomized. Level of significance was *α* = 0.05. Therefore the analyses for secondary outcomes were explorative. All tests were two-tailed. SPSS version 25 was used for data analysis.

### Role of the funding source

The funder had no role in study design, data collection, analysis, or interpretation or writing of the article. The corresponding author had access to all study data and had the final responsibility for the decision to submit for publication.

## Results

### Patients

**Fig. 1:**
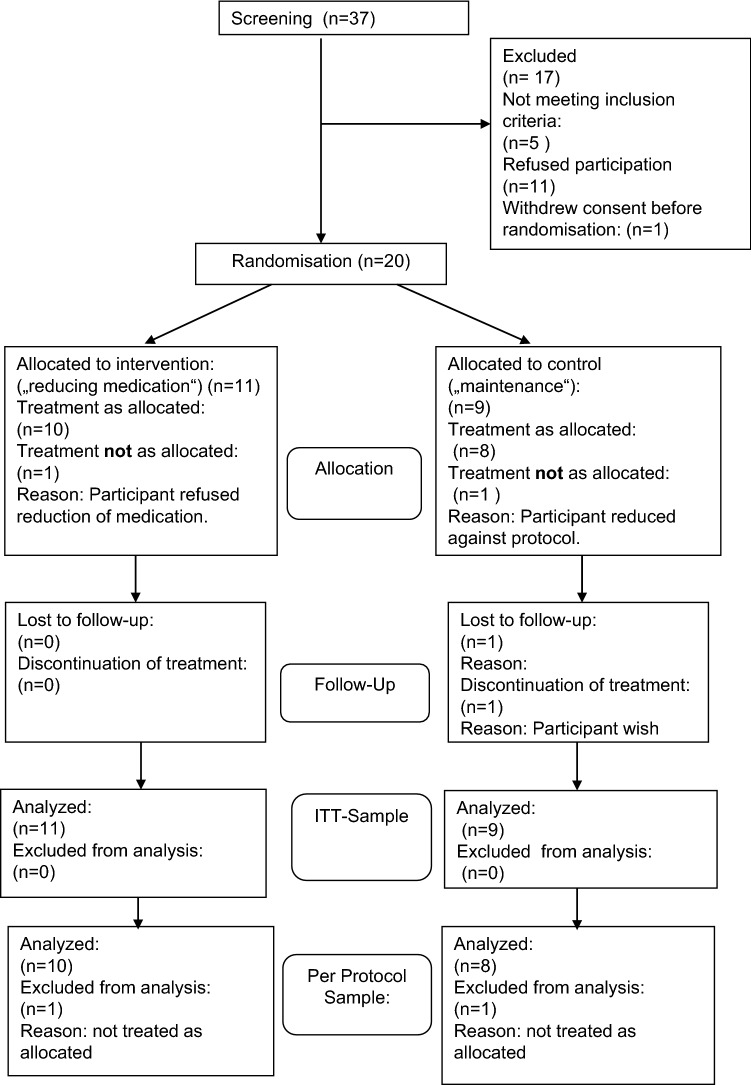
Consort-flowchart

We screened 37 patients between February 1, 2015 and December 2015 for study inclusion. Twenty-one of them were eligible and gave informed consent. One withdrew consent before randomization and was therefore not available for analysis. The initial recruitment aim was 25 patients, but the study was prematurely terminated due to lack of resources at 20 patients (80% of the planned sample). Eleven patients were randomised to the antipsychotic reduction group (intervention) and nine to the maintenance group (control). One subject in the intervention group refused to reduce medication and one in the control group reduced and stopped medication. Both were excluded from the per-protocol sample. The relapsed patient in the control group discontinued participation and was lost to follow-up (Fig. 1).

Eleven participants had a diagnosis of paranoid schizophrenia according to ICD-10 and nine, a diagnosis of schizoaffective disorder. The gender distribution was 6 males/5 females in the intervention group and 6 males/3 females in the control group. Intervention/control group had a mean age of 44.73/46.11 years, a mean duration of illness of 16.82/17.89 years, 3.36/4.56 previous episodes and no schizophrenic episode for 78.09/96.78 months. The antipsychotics in the intervention/control group prescribed were aripiprazole (*n* = 6/*n* = 1), quetiapine (*n* = 3/*n* = 5), olanzapine (*n* = 1/*n* = 3), risperidone (*n* = 5/*n* = 0), and perazine (*n* = 0/*n* = 1). In the intervention group/control group, four patients/one patient took more than one antipsychotic and the mean dose in olanzapine equivalents was 14.4 mg/9.2 mg. There was no significant difference between the characteristics of the two groups, except for family history of psychiatric illness (Table 1).

**Table 1:** Baseline characteristics of the intention to treat sample

Characteristic	Intervention (*n* = 11)		Control (*n* = 9)		*F*	Statistic	
	*m*	sd	*m*	sd		df	*p*
Age (years)	44.73	10.33	46.11	12.09	0.08	1, 18	0.785
Weight (kg)	88.70	10.95	76.88	15.29	3.66	1, 16	**0.074**
Height (cm)	175.89	5.53	174.13	7.68	0.30	1, 15	0.592
PANSS overall (baseline)	50.09	10.41	47.67	8.09	0.33	1, 18	0.575
PANSS positive-score (baseline)	9.27	2.37	10.11	2.85	0.52	1, 18	0.481
PANSS negative-score (baseline)	13.73	4.47	12.00	4.56	0.73	1, 18	0.405^a)^
PANSS Allgemein-score (baseline)	27.09	5.74	25.56	5.57	0.36	1, 18	0.554
CGI (Baseline)	2.82	0.41	3.00	0.00	1.80	1, 18	0.196^a)^
Duration of illness (years)	16.82	8.44	17.89	8.80	0.08	1, 18	0.785
Number of previous hospitalisations	3.00	1.67	3.67	3.28	0.35	1, 18	0.563
Number of previous episodes	3.36	1.75	4.56	2.83	1.34	1, 18	0.263
Duration since last episode (months)	78.09	45.82	96.78	57.28	0.66	1, 18	0.428
Gender (male/female)	6/5		6/3				0.670
Living alone (no/yes)	0/11		1/8				0.450
Married or cohabiting	7/4		3/6				0.370
Educational level (low/middle/high)	3/3/5		2/0/7				0.255^b^
Continuous occupation (no/yes)	3/8		6/3				0.175
Continuous occupation (≤ 15 h/> 15 h/full-time)	1 /3/4		1/0/2				0.509^b)^
Diagnosis (schizophrenia/schizoaffective according to ICD-10)	7/4		4/5				0.653
Smoking (no/yes)	5/6		6/3				0.406
Family history of psychiatric disorders (no/yes)	5/6		0/9				**0.038**

Continuous variables were analyzed using ANOVA. Categorical variables were analyzed with Fisher’s exact test. a = Mann–Whitney *U* test, data were not normally distributed. b = Freeman-Halton extension of Fisher´s exact test for 2 × 3 field tables. m = mean, sd = standard deviation, F = test statistic, df = degrees of freedom, *p p* value

### Primary outcome, Cox-regression and hospitalisation

Two patients in the control group and one in the intervention group fulfilled the relapse criteria (Fisher’s exact test: *p* = 0.566). The patient in the intervention group returned to starting dose after relapse, stabilized and did not fulfil the relapse criteria anymore. One relapse in the maintenance group was associated with social stress (illness of the mother of the patient). The patient stabilized over time without any change in medication. The other relapsed patient in the control group stopped medication against protocol, fulfilled relapse criteria afterwards, discontinued the study and was lost to follow-up. The median time to relapse was 88.5 days in the control group and 124 days in the intervention group (Log-rank: chi^2^ = 0.751, df = 1, *p* = 0.386) (Supplementary Fig. 1).

Using Cox-Regression to explore the influence of age, gender or PANSS-Baseline-Score on time to relapse did not yield any significant results. There was a trend for the moderator age (*p* = 0.059) (Supplementary Fig. 2), however this result was mainly caused by the youngest participant, who had the earliest relapse. This was the participant, who violated the protocol by reducing medication in the control group.

No participant had to be hospitalised during the study. Whether the one participant who dropped out was hospitalised remained unclear because he was lost to follow-up.

#### Secondary outcomes

There was no significant difference between groups in terms of the mean change from baseline to endpoint of PANSS total score and the PANSS subscores. Nor was there a significant difference between groups in terms of CGI-improvement, quality of life (SWN-Scale), social functioning (PSP), dyskinesia (AIMS), and adherence (MARS) (Table 2).

**Table 2:** Differences between baseline and endpoint in all secondary outcomes

Differences V15 − V2	Intervention			Control			Factor group		
	*n*	m/median	sd/–	*n*	m/median	sd/–	F/U	df	*p*
PANSS total^a^ (LOCF)	**11**	**− 3.45**	**10.61**	**9**	**3.00**	**9.59**	**1.55**	**1, 17**	**0.230**
*PANSS positive score*^a^* (LOCF)*	*11*	*1.0*	*–*	*9*	*0.0*	*–*	*45.5*	*1*	*0.766*
PANSS negative score^a^ (LOCF)	**11**	**− 3.82**	**3.52**	**9**	**− 0.56**	**5.29**	**1.96**	**1, 17**	**0.179**
PANSS general score^a^ (LOCF)	**11**	**− 0.55**	**6.68**	**9**	**1.33**	**2.83**	**0.16**	**1, 17**	**0.691**
SWN^b^ (LOCF)	**8**	**1.63**	**16.34**	**8**	**3.88**	**11.08**	**0.04**	**1, 13**	**0.838**
MARS sum^b^	**11**	**0.27**	**1.35**	**8**	**0.38**	**0.92**	**0.22**	**1, 16**	**0.643**
*PSP*^b^	*11*	**− ***10.0*	*–*	*8*	*10.0*	*–*	*24.0*	*1*	*0.109*
*AIMS*^a^	*11*	*0.0*	*–*	*8*	*0.0*	*–*	*41.5*	*1*	*0.840*
*CGI (LOCF)*	*Improved: n* = *3, unchanged: n* = *7, deteriorated: n* = *1*			*Improved: n* = *2, unchanged: n* = *6, deteriorated: n* = *1*			*–*	*–*	1.00*

ANCOVA for all normally distributed outcomes (bold values), Mann–Whitney *U* test for all others (italic values), except for *Freeman–Halton extension of Fisher’s exact test

*df* degrees of freedom, *PANSS* positive and negative syndrome scale of schizophrenia, *CGI* clinical global impression score (V15 vs. V2: improved/unchanged/deteriorated). SWN subjective wellbeing under neuroleptics, *MARS* medication adherence rating scale, *PSP* personal and social performance scale, *AIMS* abnormal involuntary movement scale, *m/median* mean for normally distributed and median for non normally distributed values, *n *number of participants, *sd* standard deviation, *LOCF* last observation carried forward, *V2* visit 2, *V15* visit 15

^a^Negative change scores represent improvement

^b^Positive change scores represent improvement

#### Safety

There were no serious adverse events or deaths. Side effects according to the UKU-scale are presented in Supplementary Table 1a + b. There was no significant difference between groups for any side effect at baseline or endpoint. In the course of the study, the number of patients with orgastic dysfunction decreased in the intervention group but increased in the control group (Breslow–Day test: *p* = 0.013). Dream activity increased in the intervention group but decreased in the control group (Breslow–Day test: *p* = 0.011) (details in Supplementary Table 1a + b).

#### Reduction of doses

**Fig. 2:**
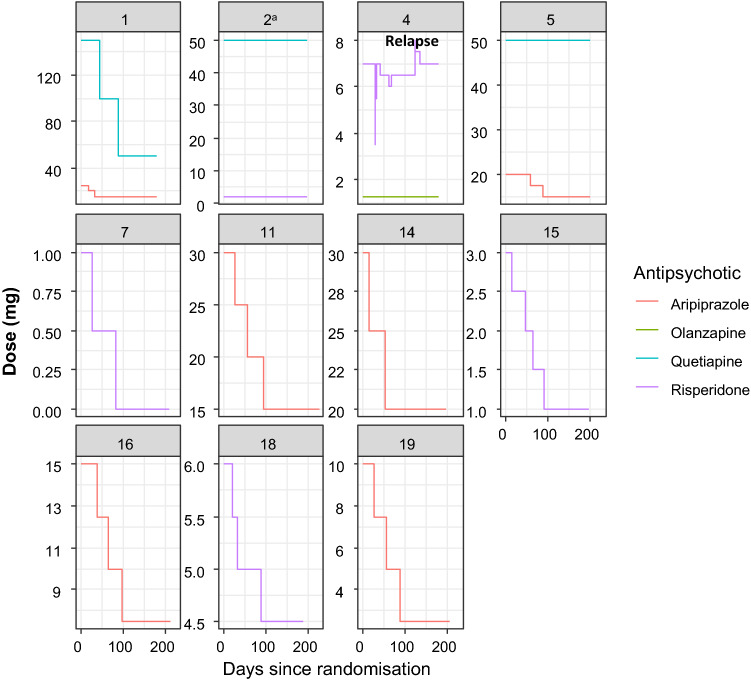
Dose reduction from baseline to endpoint in original doses for the individual subjects

The mean reduction of antipsychotic dose in the intervention group, calculated as the arithmetic mean per patient of the reduction for each antipsychotic medicament used, was 42% (range 0–100%) (Table 3), corresponding to a mean olanzapine equivalence dose of 9.5 mg/day. In one patient medication was stopped completely. Reduction schemes are presented in Fig. 2. Doses were not changed in the control group except for the patient who stopped medication himself.

**Table 3:** Reduction of medication (dosage at study end vs. baseline) in patients of the intervention group—ITT-Sample

Pat	Start medication	Start dosage (mg)	End dosage (mg)	Reduction (%)	Mean reduction per patient (%)
001	Aripiprazole	25	15	40	
	Quetiapine	150	50	66.7	53.3
002^a^	Risperidone depot	25	25		
	Quetiapine	50	50	0.0	0
004	Risperidone^b^	7	7	0^c^	
	Olanzapine	1.25	1.25	0	0
005	Aripiprazole^d^	20	15	25.0	
	Quetiapine	50	50	0.0	12.5
007	Risperidone	1	0	100.0	100.0
011	Aripiprazole	30	15	50.0	50.0
014	Aripiprazole	30	20	33.3	33.3
015	Risperidone	3	1	66.7	66.7
016	Aripiprazole	15	7.5	50.0	50.0
018	Risperidone	6	4.5	25.0	25.0
019	Aripiprazole	10	2.5	75.0	75.0
	Mean medication reduction in the intervention group				**42.3**

^a^Patient refused medication reduction

^b^Before study start patient had 1 mg oral risperidone daily and 50 mg risperidone depot every 2 weeks. Depot medication was switched to 6 mg oral risperidone

^c^After a relapse the medication had to be raised to the starting dose

^d^Patient had 400 mg aripiprazole every 4 weeks, which were switched to 20 mg oral aripiprazole daily

#### Rescue medication

The participant with the relapse in the intervention group took rescue medication (Lorazepam 1 mg) three times. Three patients in the control group took rescue medication (Lorazepam 1 mg), all due to sleeping problems and inner tension. The patient who stopped medication against protocol took rescue medication (Lorazepam 1 mg) due to craving for alcohol.

The per-protocol analysis did not materially change the results.

#### Power analysis for future studies

From the present study, a power analysis for future studies can be derived, intending to show non-inferiority of medication reduction comparing to a control group maintaining at the current dosage, when from the present study relapse rates of 15% in both the intervention and the control group are estimated. Assuming *α* = 0.05 and a power of 1 − *β* = 0.8, in both groups 26 patients will be needed to conclude that the upper limit of a one-sided 95% confidence interval will exclude a superiority in favor of the control group of more than 25% [29].

## Discussion

To our knowledge, this is the first time guided antipsychotic discontinuation was examined in stable chronic schizophrenic patients in a randomized controlled design. The goal of this pilot study was to check feasibility and safety. During the trial, no patient had to be hospitalized and the single patient with a relapse in the intervention was stable at the end of the trial after increasing his dose to the original level. One participant in the control group, who violated the study protocol by abruptly stopping medication, relapsed, dropped out and was lost to follow up. The relapse of the other patient in the control group was associated with intercurrent illness of his mother, showing that even long-term stable medicated patients are vulnerable to stress and psychosocial support is crucial for a successful reduction of antipsychotic medication [30]. The mean percentage dose reduction in the intervention group was more than 40% in three months, which is quite a substantial reduction. As intended the sample consisted of patients with chronic schizophrenia or schizoaffective disorder with several previous episodes and a mean duration of illness of 15 years (Table 1). The intention-to-treat population had a higher relapse rate in the control group than in the intervention group, but this was due to the abrupt withdrawal of a patient in the control group. In the per-protocol population one patient per group relapsed. None of the outcome measures was significantly different between the two groups. Although the sample size was small for definitive conclusions, these results make us confident that examining gradual and careful dose-reduction can be safely assessed in a future randomised trial. The only exceptions were increased dream activity and less orgastic dysfunction in the intervention group at endpoint.

There are limitations of our study. The sample was small, because this was a pilot study with the aim to check the feasibility of a larger trial. So all analyses should be seen as exploratory. The inclusion of patients with schizoaffective disorder, could have influenced the results, as there are data for a better prognosis for these patients [31]. Of the three participants who relapsed two had diagnosis of schizoaffective disorder and one a diagnosis of schizophrenia. Some participants had additional psychotropic drugs like antidepressants or mood stabilizers, which could have influenced psychopathology (Supplementary Table 2). But these medication had to be stable at least six weeks before enrollment and was not changed throughout the study. So possible changes in psychopathology cannot be attributed to these drugs. Even if clozapine is often used in chronic schizophrenic patients, we did not include this drug in our study. Our rational was evidence that reduction of clozapine can cause severe rebound psychoses [19]. Two included patients violated the protocol. These two patients did not reduce or maintain the dose according to the group they were allocated. Regardless, excluding them from the analysis did not change the results significantly. In time series analysis, the time until the first relapse was analyzed. Whether patients remitted again after they had relapsed was not an outcome of the study. However, the individual course of the patients is described in the section “Primary outcome, cox-regression and hospitalization” and indeed all patients remitted again. The goal to reduce 1/6 of the antipsychotic dose every two weeks was ambitious, but in clinical routine patients often urge doctors to reduce the dose as fast as possible. After all we assume, that a slower dose reduction could have prevented or at least delayed the one relapse in the reduction group. The follow-up period of three months after dose reduction was short and it is possible that more relapses occur in the reduction group later on. Furthermore, quality of life and social functioning, need time to change, eventually up to 7 years as seen in the study by Wunderink [32], so possible changes may be missed due to the short follow-up period. There is also contrary evidence from a ten year naturalistic follow-up of a discontinuation study [33]. In the discontinuation group, more patients had a poor clinical outcome (39%) than in the maintenance group (21%). Also the suicide rate was higher in the discontinuation group (4%) than in the maintenance group (2%).

This pilot trial showed that gradual reduction of antipsychotic medication in chronic stable schizophrenic patients is feasible and can be carefully attempted under close supervision, at least in the framework of a randomized trial with study visits every two weeks. Even patients that relapsed under dose reduction stabilized after raising medication until the end of the trial and no relapsed patient had to be hospitalized. As this was a small pilot study, results need to be confirmed in a larger trial.

## Electronic supplementary material

Below is the link to the electronic supplementary material.

Supplementary file1 (DOCX 46 kb)
