# Supplementary material for: Reducing antipsychotic drugs in stable patients with chronic schizophrenia or schizoaffective disorder: a randomized controlled pilot trial
Source: Eur Arch Psychiatry Clin Neurosci. 2020 Feb 15;271(2):293–302. doi: 10.1007/s00406-020-01109-y (PMC7960583; doi:10.1007/s00406-020-01109-y)
Supplement: Supplementary file 1 — Supplementary file1 (DOCX 46 kb) [file 406_2020_1109_MOESM1_ESM.docx]

**Appendix: Supplementary material**

|  |  | | | | | **Fisher's** |  | | | | | **Fisher's** | **Breslow-Day test** | | |
| --- | --- | --- | --- | --- | --- | --- | --- | --- | --- | --- | --- | --- | --- | --- | --- |
|  | **V1 / V2** | | | | | **Exact test** | **V15** | | | | | **Exact test** | **Factor time (V15 vs. V1/V2)** | | |
| **Side effect** | **Control N=8** | **Intervention N=8** | **Chi²** | **df** | **p^a^** | **p^b^** | **Control N=8** | **Intervention N=10** | **Chi²** | **df** | **p^a^** | **p^b^** | **Chi²** | **df** | **p^c^** |
| Concentration Difficulties | 4 | 5 | 0.3 | 1 | 0.61 | 1.00 | 3 | 2 | 0.7 | 1 | 0.41 | 0.61 | 0.8 | 1 | 0.35 |
| Astheniat/Lassitude/lncreased Fatigability | 2 | 5 | 2.3 | 1 | 0.13 | 0.31 | 2 | 7 | 3.6 | 1 | 0.058 | 0.15 | 0.0 | 1 | 0.83 |
| Sleepiness/Sedation | 3 | 4 | 0.3 | 1 | 0.61 | 1.00 | 1 | 3 | 0.8 | 1 | 0.37 | 0.59 | 0.1 | 1 | 0.72 |
| Depression | 2 | 1 | 0.4 | 1 | 0.52 | 1.00 | 1 | 4 | 1.7 | 1 | 0.20 | 0.31 | 1.8 | 1 | 0.18 |
| Failing Memory | 3 | 3 | 0.0 | 1 | 1.00 | 1.00 | 2 | 1 | 0.7 | 1 | 0.40 | 0.56 | 0.4 | 1 | 0.51 |
| Tension/lnner Unrest | 3 | 1 | 1.3 | 1 | 0.25 | 0.57 | 2 | 4 | 0.4 | 1 | 0.50 | 0.64 | 1.7 | 1 | 0.19 |
| Reduced Duration of Sleep | 2 | 1 | 0.4 | 1 | 0.52 | 1.00 | 1 | 2 | 0.2 | 1 | 0.67 | 1.00 | 0.6 | 1 | 0.45 |
| Increased Duration of Sleep | 3 | 2 | 0.3 | 1 | 0.59 | 1.00 | 3 | 3 | 0.1 | 1 | 0.74 | 1.00 | 0.0 | 1 | 0.87 |
| Increased Dream Activity | 3 | 0 | 3.7 | 1 | 0.055 | 0.20 | 1 | 5 | 2.8 | 1 | 0.094 | 0.15 | 6.5 | 1 | 0.011 |
| Emotional indifference | 1 | 2 | 0.4 | 1 | 0.52 | 1.00 | 1 | 1 | 0.0 | 1 | 0.87 | 1.00 | 0.3 | 1 | 0.58 |
| **psychiatric (total number)** | **26** | **24** | **F** | **df** | **p^d^** |  | **17** | **32** | **F** | **df** | **p^d^** |  | **F** | **df** | **p^e^** |
| **psychiatric (mean number per group)** | 3.250 | 3.000 | **0.0** | **1, 14** | **0.84** |  | 2.125 | 3.200 | **1.0** | **1, 16** | **0.32** |  | **0.7** | **1, 14.9** | **0.41** |
|  |  |  | **Chi²** | **df** | **p^a^** | **p^b^** |  |  | **Chi²** | **df** | **p^a^** | **p^b^** | **Chi²** | **df** | **p^c^** |
| Dystonia | 0 | 1 | 1.1 | 1 | 0.30 | 1.00 | 1 | 1 | 0.0 | 1 | 0.87 | 1.00 | 0.9 | 1 | 0.33 |
| Rigidity | 0 | 0 |  |  |  |  | 0 | 1 | 0.8 | 1 | 0.36 | 1.00 |  |  |  |
| Hypokinesia/Akinesia | 1 | 2 | 0.4 | 1 | 0.52 | 1.00 | 1 | 0 | 1.3 | 1 | 0.25 | 0.44 | 1.7 | 1 | 0.19 |
| Hyperkinesia | 0 | 0 |  |  |  |  | 0 | 3 | 2.9 | 1 | 0.090 | 0.22 |  |  |  |
| Tremor | 2 | 4 | 1.1 | 1 | 0.30 | 0.61 | 3 | 3 | 0.1 | 1 | 0.74 | 1.00 | 1.0 | 1 | 0.33 |
| Akathisia | 0 | 0 |  |  |  |  | 2 | 1 | 0.7 | 1 | 0.40 | 0.56 |  |  |  |
| **neurologic (total number)** | **3** | **7** | **F** | **df** | **p^d^** |  | **7** | **9** | **F** | **df** | **p^d^** |  | **F** | **df** | **p^e^** |
| **neurologic (mean number per group)** | 0.375 | 0.875 | **1.3** | **1, 14** | **0.27** |  | 0.875 | 0.900 | **0.0** | **1, 16** | **0.97** |  | **0.5** | **1, 15.9** | **0.48** |

**Supplementary Table 1a: Psychiatric and neurologic side effects according to UKU-Scale**

V1=visit 1, V2=visit 2, V15=visit 15,  ^a^Chi² test on independence of the number of the concerning UKU between the groups intervention, control; ^b^Fisher´s Exact Test (2-sided), alternative to Chi²-Test for independence, as requirements for Chi²-Test are not met (expected cell count >= 5); ^c^Breslow-Day test on homogeneity of the odds ratios over time (V2, V15); ^d^Univariate analysis of variance for the numbers of the UKUs in the /concerning field (psychical, neurological,…), ^e^Linear mixed model for the numbers of the UKUs in the concerning field , unstructured covariance matrix, interaction time x group.

|  | **V1 / V2** | | | | | **Fisher's** | **V15** | | | | | **Fisher's** | **Breslow-Day test** | | |
| --- | --- | --- | --- | --- | --- | --- | --- | --- | --- | --- | --- | --- | --- | --- | --- |
|  |  |  |  |  |  | **Exact test** |  |  |  |  |  | **Exact test** | **Factor time (V15 vs. V1/V2)** | | |
| **Side effect** | **Control N=8** | **Intervention N=8** | **Chi²** | **df** | **p^a^** | **p^b^** | **Control N=8** | **Intervention N=10** | **Chi²** | **df** | **p^a^** | **p^b^** | **Chi²** | **df** | **p^c^** |
| Accommodation Disturbances | 0 | 2 | 2.3 | 1 | 0.13 | 0.47 | 1 | 0 | 1.3 | 1 | 0.25 | 0.44 | 3.6 | 1 | 0.056 |
| Increased Salivation | 1 | 0 | 1.1 | 1 | 0.30 | 1.00 | 2 | 0 | 2.8 | 1 | 0.094 | 0.18 |  |  |  |
| Reduced Salivation | 3 | 1 | 1.3 | 1 | 0.25 | 0.57 | 1 | 3 | 0.8 | 1 | 0.38 | 0.59 | 0.0 | 1 | 0.15 |
| Diarrhoea | 1 | 0 | 1.1 | 1 | 0.30 | 1.00 | 2 | 2 | 0.1 | 1 | 0.80 | 1.00 | 0.7 | 1 | 0.41 |
| Constipation | 1 | 0 | 1.1 | 1 | 0.30 | 1.00 | 1 | 0 | 1.3 | 1 | 0.25 | 0.44 |  |  |  |
| Micturition Disturbances | 2 | 0 | 2.3 | 1 | 0.13 | 0.47 | 2 | 0 | 2.8 | 1 | 0.094 | 0.18 |  |  |  |
| Orthostatic Dizziness | 0 | 1 | 1.1 | 1 | 0.30 | 1.00 | 2 | 2 | 0.1 | 1 | 0.80 | 1.00 | 1.1 | 1 | 0.30 |
| Palpitations/Tachycardia | 1 | 0 | 1.1 | 1 | 0.30 | 1.00 | 2 | 2 | 0.1 | 1 | 0.80 | 1.00 | 0.7 | 1 | 0.41 |
| Increased Tendency to Sweating | 1 | 3 | 1.3 | 1 | 0.25 | 0.57 | 2 | 4 | 0.4 | 1 | 0.50 | 0.64 | 0.2 | 1 | 0.65 |
| **autonomous (total number)** | **10** | **7** | **F** | **df** | **p^d^** |  | **15** | **13** | **F** | **df** | **p^d^** |  | **F** | **df** | **p^e^** |
| **autonomous (mean number per group)** | 1.250 | 0.875 | **0.3** | **1, 14** | **0.59** |  | 1.875 | 1.300 | **0.5** | **1, 16** | **0.47** |  | **1.0** | **1, 13.2** | **0.33** |
|  |  |  | **Chi²** | **df** | **p^a^** | **p^b^** |  |  | **Chi²** | **df** | **p^a^** | **p^b^** | **Chi²** | **df** | **p^c^** |
| Rash | 0 | 0 |  |  |  |  | 1 | 0 | 1.3 | 1 | 0.25 | 0.44 |  |  |  |
| Photosensitivity | 0 | 1 | 1.1 | 1 | 0.30 | 1.00 | 1 | 0 | 1.3 | 1 | 0.25 | 0.44 | 2.4 | 1 | 0.12 |
| Weight gain | 1 | 2 | 0.4 | 1 | 0.52 | 1.00 | 0 | 1 | 0.8 | 1 | 0.36 | 1.00 | 0.3 | 1 | 0.56 |
| Amenorrhoea | 0 | 0 |  |  |  |  | 0 | 1 | 0.8 | 1 | 0.36 | 1.00 |  |  |  |
| Increased Sexual Desire | 0 | 0 |  |  |  |  | 0 | 1 | 0.8 | 1 | 0.36 | 1.00 |  |  |  |
| Diminished Sexual Desire | 2 | 2 | 0.0 | 1 | 1.00 | 1.00 | 3 | 1 | 1.9 | 1 | 0.16 | 0.28 | 1.0 | 1 | 0.32 |
| Erectile Dysfunction | 1 | 2 | 0.4 | 1 | 0.52 | 1.00 | 0 | 1 | 0.8 | 1 | 0.36 | 1.00 | 0.3 | 1 | 0.56 |
| Ejaculatory Dysfunction | 0 | 1 | 1.1 | 1 | 0.30 | 1.00 | 2 | 0 | 2.8 | 1 | 0.094 | 0.18 | 3.6 | 1 | 0.057 |
| Orgastic Dysfunction | 0 | 3 | 3.7 | 1 | 0.055 | 0.20 | 2 | 0 | 2.6 | 1 | 0.11 | 0.21 | 6.2 | 1 | 0.013 |
| Dry Vagina | 1 | 1 | 0.0 | 1 | 1.00 | 1.00 | 2 | 0 | 2.6 | 1 | 0.11 | 0.21 | 1.6 | 1 | 0.21 |
| Headache | 3 | 2 | 0.3 | 1 | 0.59 | 1.00 | 1 | 3 | 0.8 | 1 | 0.38 | 0.59 | 1.0 | 1 | 0.31 |
| **other (total number)** | **8** | **14** | **F** | **df** | **p^d^** |  | **12** | **8** | **F** | **df** | **p^d^** |  | **F** | **df** | **p^e^** |
| **other (mean number per group)** | 1.000 | 1.750 | **0.8** | **1, 14** | **0.40** |  | 1.500 | 0.800 | **1.3** | **1, 16** | **0.28** |  | **3.7** | **1, 14.1** | **0.076** |
| **Total** | **47** | **52** |  |  |  |  | **51** | **62** |  |  |  |  |  |  |  |

**Supplementary Table 1b: Autonomous and other side effects according to UKU-Scale**

V1=visit 1, V2=visit 2, V15=visit 15,  ^a^Chi² test on independence of the number of the concerning UKU between the groups intervention, control; ^b^Fisher´s Exact Test (2-sided), alternative to Chi²-Test for independence, as requirements for Chi²-Test are not met (expected cell count >= 5); ^c^Breslow-Day test on homogeneity of the odds ratios over time (V2, V15); ^d^Univariate analysis of variance for the numbers of the UKUs in the concerning field (psychical, neurological,…), ^e^Linear mixed model for the numbers of the UKUs in the concerning field, unstructured covariance matrix, interaction time x group

|  | n | Timepoint 1 | Timepoint 2 | Median |
| --- | --- | --- | --- | --- |
| **Intervention** | 1 | 124 days |  | 124 days |
| **Control** | 2 | 56 days | 121 days | 88.5 days |

**Supplementary Figure 1:** Median time until relapse (Kaplan Meier Survival Functions)

n=number of relapses

| **Omnibus Tests of Model Coefficients^a^** | | | | | | | | | |
| --- | --- | --- | --- | --- | --- | --- | --- | --- | --- |
| -2 Log Likelihood | Overall (score) | | | Change From Previous Step | | | Change From Previous Block | | |
|  | Chi-square | df | Sig. | Chi-square | df | Sig. | Chi-square | df | Sig. |
| 80.624 | 4.730 | 4 | .316 | 4.359 | 4 | .360 | 4.359 | 4 | .360 |
| a. Beginning Block Number 1. Method = Enter | | | | | | | | | |
| **Variables in the Equation** | | | | | | |  |  |  |
|  | B | SE | Wald | df | Sig. | Exp(B) |  |  |  |
| Group | .370 | .522 | .504 | 1 | .478 | 1.448 |  |  |  |
| Age (years) | -.060 | .032 | 3.571 | 1 | **.059** | .941 |  |  |  |
| Gender | -.735 | .569 | 1.664 | 1 | .197 | .480 |  |  |  |
| PANSS total (baseline) | .046 | .032 | 2.063 | 1 | .151 | 1.047 |  |  |  |

Scatterplot age x days until relapse/endpoint

**Suppplementary Figure 2:** Results of Cox regression model for relapse

| **ID*** | **group** | **diagnosis** | **relapse** | **antipsychotic** | **dose in mg** | **dose in olanzapine  equivalents in mg** | **comedication** | **dose in mg** | **indication for comedication** |
| --- | --- | --- | --- | --- | --- | --- | --- | --- | --- |
| 1 | intervention | schizophrenia | no | aripiprazole quetiapine | 25 150 | 17 4 | citalopram | 40 | depressive symptoms |
| 2 | intervention | schizophrenia | yes | risperidone quetiapine | 2 50 | 7 1 | none | na | na |
| 3 | control | schizophrenia | no | aripiprazole | 5 | 3 | lithium | 675 | mood stabilizer |
| 4 | intervention | schizoaffective disorder | yes | risperidone | 7 | 23 | Citalopram | 10 | depressive symptoms |
| 5 | intervention | schizophrenia | no | aripiprazole quetiapine | 20 15 | 13 1 | none | na | na |
| 6 | control | schizoaffective disorder | no | quetiapine | 250 | 7 | pregabalin fluoxetine | 450 40 | neuropathic pain depressive symptoms |
| 7 | intervention | schizophrenia | no | risperidone | 1 | 3 | none | na | na |
| 8 | control | schizophrenia | no | quetiapine perazine | 700 100 | 19 3 | none | na | na |
| 10 | control | schizoaffective disorder | no | olanzapine | 10 | 10 | lithium | 1350 | mood stabilizer |
| 11 | intervention | schizoaffective disorder | no | aripiprazole | 30 | 20 | venlafaxine | 300 | depressive symptoms |
| 12 | control | schizoaffective disorder | no | olanzapine | 5 | 5 | none | na | na |
| 13 | control | schizoaffective disorder | yes | olanzapine | 7,5 | 8 | zopiclone | 7,5 | sleeping disturbances |
| 14 | intervention | schizoaffective disorder | no | aripiprazole | 30 | 20 | none | na | na |
| 15 | intervention | schizophrenia | no | risperidone | 3 | 10 | valproic acid | 1250 | mood stabilizer |
| 16 | intervention | schizophrenia | no | aripiprazole | 15 | 10 | none | na | na |
| 17 | control | schizoaffective disorder | no | quetiapine | 150 | 4 | none | na | na |
| 18 | intervention | schizoaffective disorder | no | risperidone | 6 | 20 | venlafaxine | 300 | depressive symptoms |
| 19 | intervention | schizophrenia | no | aripiprazole | 10 | 7 | none | na | na |
| 20 | control | schizophrenia | no | quetiapine | 800 | 22 | none | na | na |
| 21 | control | schizophrenia | no | quetiapine | 200 | 5 | venlafaxine | 150 | depressive symptoms |

**Supplementary table 2:** Diagnosis, antipsychotic dose and comedication of the sample

The table presents additional characteristics of the sample; na=not applicable; *=participant with ID 9 withdrew consent after baseline.
